# GAD65-positive autoimmune-associated epilepsy presenting with Ictal Hand Kissing; an uncommon presentation of a rare disease

**DOI:** 10.1016/j.ebr.2025.100801

**Published:** 2025-07-01

**Authors:** Alia M.R. Fallatah, Hanan M. Abdulmutali, Majed H. Alhameed

**Affiliations:** National Neuroscience Institute, King Fahad Medical City, Riyadh, Saudi Arabia

**Keywords:** GAD65, Autoimmune associated epilepsy, Temporal lobe epilepsy, Ictal kissing, Seizure, Semiology

## Abstract

Glutamic acid decarboxylase-65 (GAD65) is an enzyme responsible for the production of gamma-aminobutyric acid (GABA). Elevated levels of GAD65 antibodies have been recognized as a key biomarker of neurological autoimmune disorders, particularly autoimmune-associated epilepsy (AAE). We report the case of a 48-year-old woman with drug-resistant right temporal AAE, who initially developed seizures following a febrile illness accompanied by confusion. Notably, the patient exhibited rare ictal semiology involving stereotypical Ictal Hand kissing (IHK) behavior. Brain MRI revealed bilateral mesial temporal sclerosis (MTS), which was more pronounced on the right side. High serum titers of GAD65 antibodies further supported this diagnosis. This case underscores the incompletely elucidated clinical features of GAD65-positive AAE, and highlights the unique semiology of IHK behavior and its role in understanding and characterizing possible underlying epileptogenic networks.

## Highlights

• Seizure is a key symptom of GAD65-AE.
• GAD65-AAE commonly originates from the temporal lobe.
• GAD65-AAE can present with rare seizure semiology.
• Ictal hand kissing is a complex behaviour of an unrevealed network.

## 1 Introduction

Glutamic acid decarboxylase-65 (GAD65) is a key enzyme responsible for producing gamma-aminobutyric acid (GABA), the main inhibitory neurotransmitter in the brain. High-titer GAD65 antibodies are vital biomarkers for diagnosing autoimmune neurological disorders including autoimmune encephalitis (AE) [1]. GAD65-associated autoimmune encephalitis (GAD65-AE) presents with various clinical features, although seizures are its hallmark symptom. Acute symptomatic seizures occur in 10–50 % of patients, with over 80 % at a risk of developing autoimmune-associated epilepsy (AAE).

Recent studies have emphasized that epilepsy is often central to GAD65-related neurological autoimmunity [2]. Budhram et al. reported that in a group of 212 patients with GAD65-AE, 29 % had epilepsy as the primary feature, predominantly originating from the temporal lobe [3]. In this report, we present a case of anti-GAD65 antibody-positive AAE, in which the patient exhibited a rare form of seizures involving stereotypical Ictal Hand kissing (IHK) behavior.

This case underscores the clinical features particular to GAD65-positive AAE and IHK behavior as a unique semiology, adding to the current understanding and characterization of possible epileptogenic networks presenting with this phenomenon. This distinctive clinical presentation, along with the specific antibody findings, highlights the importance of recognizing atypical seizure patterns and their broader implications for understanding autoimmune epilepsy.

## 2 Case presentation

### 2.1 Clinical presentation

A 48-year-old right-handed woman first began experiencing seizures at the age of 38 years following a febrile illness. In December 2012, after 5 days of fever and vomiting, she presented with confusion and noticeable changes in behavior. Shortly thereafter, she experienced generalized tonic–clonic seizures, which continued on a monthly basis. At that time, her neurological examination findings were mostly normal, except for some disorientation. Analysis of her CSF, including assessment for glucose and protein levels and cell counts, yielded normal results. The initial diagnosis was acute confusion, likely due to viral encephalitis, for which the patient was treated empirically. Although the acute confusion has resolved over subsequent weeks, she continued to struggle with memory problems and poorly controlled seizures.

At the tie of presentation, the patient experienced focal preserved consciousness seizure or focal impaired consciousness seizure with observable manifestations. These episodes were typically preceded by an aura of nausea or vomiting, followed by oral and right-hand automatism. Occasionally, she retained her ability to speak during the seizures. Seldom, she experienced brief episodes of impaired consciousness lasting only 3–5 s. Despite receiving appropriate doses of levetiracetam, lamotrigine, and lacosamide, her seizures remained drug-resistant. She had a medical history of systemic lupus erythematosus (SLE) and hypothyroidism. Additionally, she had a family history of autoimmune conditions, including SLE and rheumatoid arthritis in her sister and SLE in her maternal aunt.

During admission to the epilepsy monitoring unit (EMU), her neurological findings remained largely unremarkable, except for perioral tremor noticeable when she talks or moves her mouth in addition to low amplitude high frequency of approximately 6–8 Hz resting and kinetic tremors predominantly involving distal upper limbs bilaterally. These tremors were exaggerated with arm extension and intention. In addition, these tremors were clearly exacerbated by emotional stress, anxiety and at the onset of seizures, and were less obvious in the postictal state and while the patient was relaxed or calm.

### 2.2 Video-EEG

**Fig. 1:**
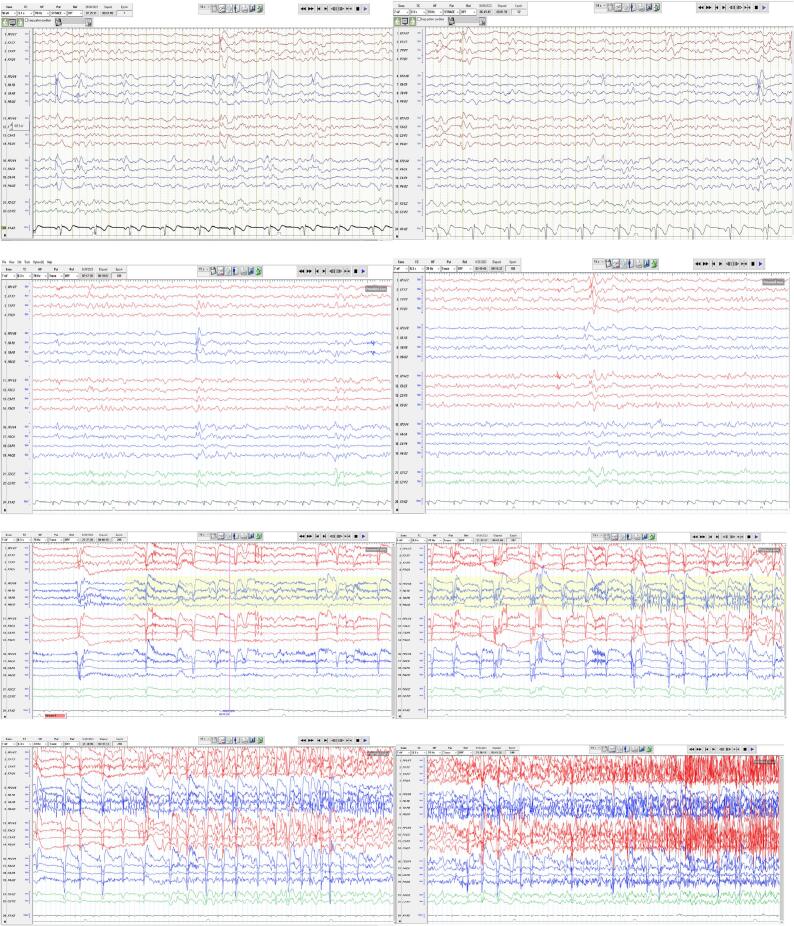
A, b, c, d: Interictal epileptiform discharges in bilateral temporal lobes. e, f, g, h: Ictal event originating from the right temporal lobe.

In September 2023, the patient was admitted to the EMU for further assessment. A 4-day video-EEG recording revealed normal background activity, interrupted by intermittent slowing and interictal epileptiform discharges in both temporal lobes, with greater prominence on the right side (Fig. 1 a, b, c, d). During the monitoring period, seven habitual seizures originating from the right temporal lobe were recorded (Fig. 1e, f, g, h). Clinically, these seizures began with an aura of nausea, followed by oral automatisms including chewing, along with kissing and spitting behaviors in some events. Other features included right hand automatism followed by clonic jerks, eyes flickering, eyes and head turning to the left side, preserved speech during certain events, and occasional loss of consciousness. On EEG, all seizures started in the right posterior temporal region (T8P8) with rhythmic theta activity which spread anteriorly then evolved to higher voltage rhythmic delta and involved the entire right hemisphere. Rarely, right temporal theta waves evolved into spike and wave complexes. A single seizure led to a bilateral tonic clonic seizure.

### 2.3 MRI

**Fig. 2:**
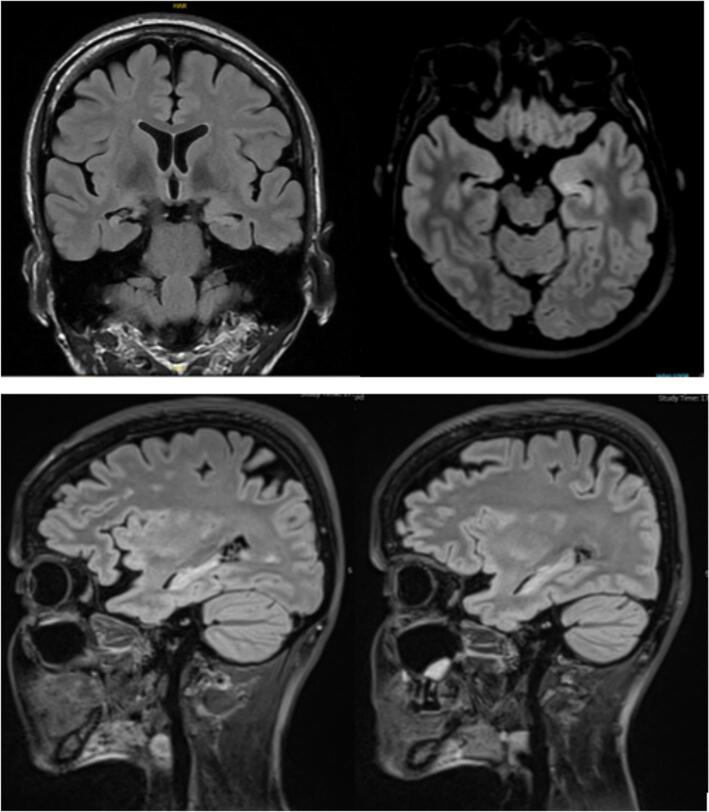
Coronal, axial and sagittal cuts of MRI T2 FLAIR showing bilateral mesial temporal sclerosis more pronounced on the right side.

Brain MRI revealed signal alteration and atrophy suggestive of bilateral mesiotemporal sclerosis (MTS), which was more pronounced on the right side than on the left side (Fig. 2).

### 2.4 Autoimmune/paraneoplastic workup

During the EMU admission in September 2023, CSF analysis revealed normal protein and glucose levels and cell counts. Serum tests showed significantly elevated GAD65 antibody titer at 445.2 IU/mL (reference range: <10.0 IU/mL). Moreover, screening for malignancies yielded negative results.

### 2.5 Management and follow up

During hospitalization, the patient was administered intravenous immunoglobulin. However, at 2- and 4-months follow-ups, she reported minimal improvement in her seizure frequency.

## 3 Discussion

### 3.1 GAD65 AAE

Low serum GAD65 levels can sometimes be detected in healthy individuals, making high titers specific for neurological autoimmunity [4]. High-titer GAD65 antibodies in serum and CSF are significant biomarkers for autoimmune neurological diseases, including stiff person syndrome, cerebellar ataxia, limbic encephalitis (LE), and AAE. GAD65-AE is increasingly being recognized as a cause of drug-resistant epilepsy (DRE), particularly temporal lobe epilepsy, often manifesting with various semiological features [3].

In contrast to patients with acute symptomatic seizures, some patients with immune-mediated brain diseases have seizures that become chronic and prove resistant to both antiseizure medications (ASMs) and immunotherapy. This has been reported frequently with GAD65 [[5], [6], [7]]. In the context of positive GAD65, the low rates of seizure freedom despite immunotherapy suggest that the risk of further seizures over the next 10 years is very high which is similar to the timespan and the disease course of our patient fulfilling the conceptual and practical definitions of epilepsy, in addition to the definition of AAE evident by high titer serum GAD65 antibodies [5,8,9].

Our patient presented with DRE, memory and cognitive impairment as well as tremor which was not described previously as part of the classical anti–GAD65 associated syndromes or the overlap syndrome [1]. Tremor may be an incidental co-occurrence or it could be part of the patients presenting symptoms constellation; however, the exact cause remains unknown. This emphasizes a possible wider range of under reported symptoms and signs of GAD65 related neurological autoimmunity. We found a single report of pseudo-orthostatic tremor as a phenotype in presence the of high-titer GAD65, however that is another entity and the full clinical picture was not clearly described [1].

Classically, the patient’s serum GAD65 antibody titers were high and she had MRI evidence of bilateral MTS. Additionally, her EEG showed seizures originating from the right posterior temporal region. Moreover, she exhibited other features of GAD65 AAE such as seizure onset after LE, history of other autoimmune conditions, family history of autoimmunity and poor response to ASMs and immunotherapy.

Epilepsy, with or without LE, is a core feature of GAD65-associated neurological autoimmunity [10]. Temporal lobe epilepsy is the most common presentation of GAD65-AE, occurring in 29 % of patients. Among them, 63 % experience medial temporal lobe-onset seizures [3]. These seizures often display temporal-perisylvian characteristics. Other areas of seizure onset include the frontal, temporoparietal, and temporooccipital regions, highlighting the varied nature of autoimmune epilepsy [3,11].

As evident in our case, seizures associated with GAD65 neurological autoimmunity are frequently refractory to medical treatment, with 74 % of the patients not responding to standard ASMs [3]. Furthermore, GAD65-AE is less likely to be associated with malignancy compared to other autoimmune encephalopathies. Cognitive impairment, particularly short-term memory and attention, is often a secondary manifestation, which underscores the broad neurological impact of the condition [3,12]. Although our patient didn’t have a formal neuropsychological assessment, her cognitive impairment was clearly indicated by history and noticed during clinical assessment. She works as a school teacher and she reported continuing carrying out her educational duties with difficulties attributed to memory and concertation challenges.

### 3.2 Ictal Hand Kissing

A particularly intriguing aspect of GAD65-related epilepsy is its association with unusual ictal behaviors. Our patient exhibited a rare seizure semiology known as Ictal kissing (IK). This behavior is uncommon. It has been documented in cases of non-dominant temporal lobe epilepsy, often linked with hippocampal sclerosis [[13], [14], [15], [16]].

Kissing is described as a motor act of puckering the upper and the lower lips together to kiss oneself, someone else or blowing the kiss in the air. This behavior has complex motor and emotional components which makes it difficult to consider it as a simple automatism.

**Fig. 3:**
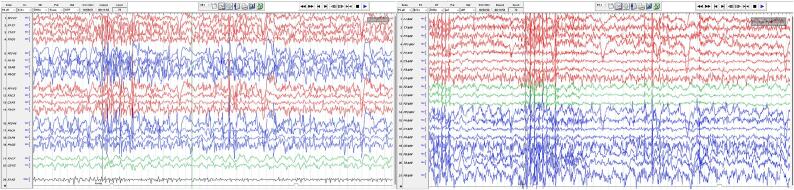
A bipolar and b referential montages demonstrating a seizure evolving over the right hemisphere. the onset of ik was clearly preceded by ictal spread to the frontal region on EEG.

IK typically involves a specific motor pattern. Our patient, had stereotypical IHK. The motor component involved the lips, right upper limb and truck turning to the right in addition to articulation for requesting the watcher hand. During each seizure, she requested the observer’s hand, turned her head and torso to the right, and kissed the hand. On EEG, seizures started in the posterior temporal area then spread over the right hemisphere (Fig. 3).

Although the behavioral aspect is poorly understood, we could easily appreciate on video-EEG that the patient was experiencing an emotional change. She seemed anxious and agitated while waiting for her observe to reach out, however, it is hard to identify what she felt and the emotional component of this behavior remains unclear due to postictal amnesia, despite the patient’s preserved consciousness during seizures.

It was previously suggested that the genesis of IK reflects the involvement of the limbic system and activation and/or inhibition of Temporo-frontal network [17]. Another postulated theory behind IK is the Carillon theory which suggests that A key aspect of the neurobiological underpinnings of IK is the involvement of central pattern generators in the brainstem and spinal cord that regulate essential survival behaviors such as feeding, locomotion, and reproduction [14,18].

## 4 Conclusion

This report highlights the uncommon occurrence of IHK in GAD65-positive AAE and provides valuable insights into the existing literature. By documenting this case, we aimed to enhance our understanding of GAD65 positivity impact on autoimmune epilepsy and its prognostic implications particularly regarding seizure freedom, response to ASMs and immunotherapy providing a foundation for future therapeutic considerations. Furthermore, reporting IHK behavior, provide insights into seizure localization, underlying networks and emphasize the importance of recognizing atypical semiologies.

## Ethics approval statement

The study was approved by the Institutional Review Board of King Fahad Medical City, Riyadh Second Health Cluster on December 12, 2023. IRB Log Number: 23-664”.

IRB Registration Number with KACST, KSA: H-01-R-012.

IRB Registration Number with OHRP/NIH, USA: IRB00010471.

Approval Number Federal Wide Assurance NIH, USA: FWA00018774.

## CRediT authorship contribution statement

**Alia M.R. Fallatah:** Writing – review & editing, Writing – original draft, Data curation, Conceptualization. **Hanan M. Abdulmutali:** Writing – review & editing, Data curation. **Majed H. Alhameed:** Writing – review & editing, Resources, Conceptualization.

## Declaration of competing interest

The authors declare that they have no known competing financial interests or personal relationships that could have appeared to influence the work reported in this paper.
